# 

**Published:** 1905-12-02

**Authors:** 


### NOTICE TO CORRESPONDENTS.

All MSS., Letters, Books for Review, and other matters intended for the Editor should be addressed The Editor, "The Hospital," The Hospital Buildings, 28 & 29 Southampton Street, Strand, W.C.

The Editor cannot undertake to return rejected MS., even when accompanied by stamped directed envelope.

**Notice to Advertisers and Subscribers.**

All Advertisements, Orders for Copies of The Hospital, and Business Communications should be addressed to **THE MANAGER (Not to the Editor),** The Hospital, 28 & 29 Southampton Street, Strand, London, W.C.

The Cost of Subscription, post free, is at follows:—Per week, 3½d.; per quarter, 3s. 3d.; per half-year, 6s. 6d.; per annum, 13s. Foreign and Colonial:—Per annum, 19s. 6d., payable, in all cases, in advance.

**NOTICE.—Vol. XXXVIII. of "The Hospital" April to September 1905), in handsome cloth binding, will shortly be on sale at the office of this Journal, price 10s. 6d. Cases for binding the Numbers contained in this Volume 2s. Index to Vol. XXXVIII., 3½d., post free.]**

**The Monthly Part for December is now ready. Price 1s., by post, 1s. 3d.**

### MEDICAL APPOINTMENTS.

**James Grant Andrew,** M.B., M.S.Glasg., Surgeon to the new wards at the Victoria Infirmary, Glasgow. **R. J. Archibald,** M.R.C.S., L.R.C.P.Lond., Clinical Assistant to the Chelsea Hospital for Women, Fulham Road, London, S.W. **Arthur H. Burgess,** F.R.C.S.Eng., M.B., M.Sc.Vict., Honorary Assistant Surgeon to the Manchester Royal Infirmary. **Donald Campbell,** M.D., C.M.Glasg., Medical Officer of Health of Calne (Wilts). **Theodore Fisher,** M.D.Lond., M.R.C.P.Lond., M.R.C.S., on the Honorary Medical Staff of the East London Hospital for Children, Shadwell. **Ernest J. C. Groves,** M.B., Ch.B.Edin., House Surgeon at the Swansea General and Eye Hospital. **Frank Hewkley,** M.B.Durh., F.R.C.S.Eng., Clinical Assistant to the Western Ophthalmic Hospital. **John Mullin,** L.R.C.P.& S.Edin., Medical Officer of Health of Strokestown, Ireland. **J. Sidney Pearson,** M.B.Cantab., M.R.C.S., L.R.C.P., Senior Resident Medical Officer at St. Mary's Hospital for Women and Children, Plaistow, E. **Arthur Rendle Short,** M.D., B.S., B.Sc.Lond., House Surgeon at the Bristol Royal Infirmary. **H. Hammond Smith,** M.R.C.S., L.R.C.P.Lond., a Medical Referee under the Workmen's Compensation Acts, 1897 and 1900, and to act for Brentford, Brompton, and Marylebone County Courts in County Court Circuit No. 43. **J. C. Teasdale,** M.B., Ch.B.Vict., M.R.C.S.Eng., Surgeon to the Retford Hospital. **Richard Warren,** M.D., B.Ch.Oxon., F.R.C.S.Eng., L.R.C.P.Lond., Senior House Surgeon at Poplar Hospital for Accidents. **James Weir,** M.B., M.S.Glasg., Assistant Surgeon to the new wards at the Victoria Infirmary, Glasgow. **M. T. Williams,** M.B., B.S.Lond., F.R.C.S.Eng., Honorary Physician to the Kent and Canterbury Hospital.

St. Thomas's Hospital.—The following gentlemen have been selected as house officers at St. Thomas's Hospital:—Casualty Officers (from January 1st, 1906): **H. A. Kisch,** M.R.C.S., L.R.C.P. (*Senior*); **G. R. Footner,** M.B., B.C.Cantab., M.R.C.S., L.R.C.P. (*Junior*). Resident House Physicians: **G. J. Langley,** M.B., B.S.Lond., (*Extension*); **C. L. Morgan,** M.B., B.S.Lond., M.R.C.S., L.R.C.P. (*Extension*); **W. O. Meek,** M.R.C.S., L.R.C.P.; **C. St. A. Coles,** M.R.C.S., L.R.C.P. House Physicians to Out-Patients: **F. M. Bulley,** M.R.C.S., L.R.C.P., M.A.Cantab.; **R. C. Jewesbury,** M.R.C.S., L.R.C.P., B.A.Oxon. Resident House Surgeons: **L. E. C. Norbury,** M.B., B.S.Lond., M.R.C.S., L.R.C.P. (*Extension*); **D. K. Coutts,** M.B., B.S.Lond., M.R.C.S., L.R.C.P. (*Extension*); **J. H. Bletsoe,** M.B., B.S.Lond., M.R.C.S., L.R.C.P. (*Extension*); **R. J. C. Thompson,** M.R.C.S., L.R.C.P. (*Extension*). House Surgeons to Out-Patients: **H. T. Gray,** B.A. Cantab., M.R.C.S., L.R.C.P. (*Extension*); **A. W. Hooker,** M.B., B.S.Lond., M.R.C.S., L.R.C.P. (*Extension*); **J. H. Drew,** M.B., B.S. Lond., M.R.C.S., L.R.C.P. (*Extension*); **H. Falk,** B.A.Cantab., M.B., B.C., M.R.C.S., L.R.C.P. (*Extension*). Obstetric House Physicians: **J. M. Wyatt,** M.B., B.S.Lond., M.R.C.S., L.R.C.P. (*Senior*); **R. E. Whiting,** M.A., M.B. B.C.Cantab. (*Junior*). Ophthalmic House Surgeons: **F. R. E. Wright,** M.B.Lond., M.R.C.S., L.R.C.P., D.P.H. (*Senior*); **C. R. B. Eyre,** M.R.C.S., L.R.C.P. (*Junior*). Special Departments: (Throat) **R. H. Cotton,** M.R.C.S., L.R.C.P., (*Extension*); **F. S. Hewett,** M.R.C.S., L.R.C.P., B.A.Cantab. (Skin) **W. C. A. Ward,** M.R.C.S., L.R.C.P. (*Extension*); **A. B. Howitt,** M.R.C.S. L.R.C.P., B.A.Cantab. (Ear) **H. S. Sington,** M.R.C.S., L.R.C.P. (*Extension*); **F. D. Atkins**' M.R.C.S., L.R.C.P. (*Extension*).

### A New and Valuable Addition to Nursing Literature.

**Ready Next Week.**

### SURGICAL INSTRUMENTS AND . . APPLIANCES USED IN OPERATIONS

**1/6** net, **1/8** post free.

**An Illustrated and Classified List, with Explanatory Notes.**

**1/6** net, **1/8** post free.

By HAROLD BURROWS, M.B. (Lond.), F.R.C.S.,
Assistant Surgeon to Bollingbroke Hospital.

**This book should prove of the utmost assistance to those upon whom the duty of making arrangements for a surgical operation may fall. The list given represents as nearly as possible what may be regarded as the average requirements, and the Nurse will not go far wrong in following them to the letter. The fact, too, that the work is very fully illustrated renders it of great help in identifying the various appliances.**

***N.B.—The demand for this book is already so large that Nurses requiring copies should order immediately, as the First Edition is limited and will soon be exhausted.***

How to Succeed at the C.M.B. Exam.

**A COMPLETE HANDBOOK OF MIDWIFERY.**

By J. K. WATSON, M.D. 6/- net, 6/4 post free.
Profusely Illustrated with 143 Diagrams.

This book has been **compiled in accordance with the requirements of the Central Midwives Board,** and will prove not only the best text-book for those entering for the next exam., but is so complete as to be of greatest value to the Qualified Midwife for immediate reference in cases of emergency.

The Midwives Act Explained.

**HOW TO BECOME A CERTIFIED MIDWIFE.**

By Dr. APPEL. 1/- net; 1/1 post free.

This book gives a very clear and complete explanation of the Midwives Act, and a copy should be in the possession of all Midwives and those nurses desirous of taking up the special branch.

**INSTRUCTIONS TO MIDWIVES.**

Compiled by the Manchester Midwives' Committee.
Crown 8vo. cloth, **6d.** net, **7d.** post free.

Midwives will find this little work exceedingly useful as it explains simply and concisely what should be done in accordance with the rules of the Midwives Act.

Medical Electricity.

**MEDICAL ELECTRICITY AND THE LIGHT TREATMENT.**

By KATE NEALE,
Sister in Charge Light Department, Guy's Hospital.
Cloth, profusely Illustrated.
**2/6** net, **2/9** post free.

A practical book on a subject hitherto untouched from a nursing standpoint.

The Duties of a Nurse Explained.

**It is essential that anyone desirous of taking up Nursing should have some idea of the duties of a Nurse.**

**NURSING HINTS TO PROBATIONERS ON PRACTICAL WORK.**

By ANNESLEY VOYSEY. Price **2/-** net, **2/4** post free.

**Contains full particulars of a Nurse's duties, and will prove of the greatest assistance to the Probationer.**

An Important New Work on Sanitation for Nurses.

**SIMPLE SANITATION:**
**The Practical Application of the Laws of Health to Small Dwellings.**

By M. LOANE,
Superintendent of District Nurses, Portsmouth.
Limp cloth, **1/-** net, **1/2** post free.

With an Introductory Chapter on Sanitary Legislation by Dr. A. MEARNS FRASER, Medical Officer of Health, Portsmouth.

This work should prove of great practical assistance to Midwives and District Nurses, as it contains Chapters on Sanitary Legislation, Water, Air and Ventilation, Drainage and Disposal of Refuse, Household Management, Personal Hygiene, Disinfectants, &c.

Nursing of Children.

**NURSING OF SICK CHILDREN.**

By Dr. BURNET. **1/-** net, **1/1** post free.

Nurses and Mothers will find this a most useful handbook.

***Complete Catalogue sent post free on application.***

**The Scientific Press, Ltd.**

**Publishers of Nursing Text-Books, Charts, and Case-Books of all Descriptions.**

**28 & 29 SOUTHAMPTON STREET, STRAND, LONDON, W.C.**

